# Preclinical tests in Brazil

**DOI:** 10.1186/1753-6561-8-S4-O33

**Published:** 2014-10-01

**Authors:** Ingrid Taricano

**Affiliations:** 1 Universidade Nove de Julho - UNINOVE, Água Branca, SP, Brazil

## 

Historically the Brazilian pharmaceutical industry went through different phases, which reflected, at least in part, national economic stages. Until the last decade Brazil was dominated by foreign pharmaceutical industry. This has been changing as some national companies, supported by the federal government, have started developing new molecules derived from academic research, as well as producing drugs whose patents have expired. Biopharmaceuticals are extremely sophisticated drugs, synthesized by cell manipulation and designed to treat complex diseases. This change in the industry's attitude pointed to new requirements, well known in developed countries, such as greater regulatory involvement, training people, structuring specialized centers, among others. Naturally, some centers have emerged in the last two decades and these changes have brought a new impetus to preclinical studies laboratories. This fact was evidenced by Ordinance No. 8 (of 16th June 2011), which established the Working Group for the articulation of Reference Centers in Pharmacology. Furthermore, other actions were also reinforced, such as the introduction of Good Laboratory Practice (GLP) by INMETRO, the search of bilateral recognition for toxicology studies between Brazil and OECD, the release of the Guidelines for nonclinical security studies required for drug development by ANVISA, and the establishment of the regulatory agency for animal facilities (Conselho Nacional de Controle de Experimentação Animal - CONCEA), created in 2008 with the approval of the "Arouca" Law. ANVISA, however, still has a timid performance in pre-clinical studies. It is based on the law of September 23, 1976, which addresses the need to prove the drug efficacy and safety, and the RDC No 136 of May 29, 2003, which mentions the need for reporting preclinical trials results for a new drug to be registered.

At this point, it should be emphasized that there is a long way to go, considering that for risk and safety assessment of new drugs (especially as it relates to Biopharmaceuticals) there is often a need to apply two or three concomitant guidelines including EMEA, ICH and OECD for designing a study plan. This practice also brings with it the need for training people in the scope of quality, as well as management of this practice. Depending on their size and strategy, multinational pharmaceutical companies may conduct extensive in-house research or seek to license promising drugs from academia, other pharmaceuticals, or biotechnology companies. The Brazilian academic institutions have substantial research and publications related to new molecules, as well as new therapeutic applications for known molecules. However the practice of partnerships between the academia and industry is quite new in our country. Few industries have learned this path as a route to innovation, but those who have already tried it, are emerging as innovative companies obtaining their first products.

Following the development of new drugs, preclinical studies stay in between the academic research and the clinical trials, to determine the safe use interval between the effectiveness and the therapeutic regimen. Thus, it is not uncommon that a long and laborious period is devoted to this phase, since the data obtained in the development of protocols may require changes in doses, in the molecule, in the pharmaceutical formulation or in the vehicle. Thus, the need for a well trained staff, working as a team, with knowledge of regulatory compliance practices (GLP) and good relationships with the industry that owns the investigated molecule is a must. A solid development requires the participation of leading scientists in each one of the areas involved in the discussion of the data obtained. These professionals are veterinarians, professionals of veterinary clinical analysis lab, veterinary pathologists with training in toxicology, as well as the toxicologist as the director of studies in accordance with BLP. Correct decisions about the course of development of the drugs are based on such discussions, which should also include toxicologists from the industry staff, who must have the ability to understand the importance of data and guide the necessary changes. The interaction between the laboratories that develop the preclinical protocols and the industry, tends to promote common learning, generating aggregation of skills to the research area and the overall development of the pharmaceutical industry. It is important to note in this regard that most Brazilian companies are still in this learning process and have not yet internalized all the requirements discussed above. This is particularly so for biopharmaceuticals, for which only recently ANVISA has opened applications for registration.

The gradual and progressive structuring of the area of preclinical trials in Brazil, according to the standards described above, favors the expansion and strengthening of the Brazilian pharmaceutical industry, opening opportunities to external marketing of our products in addition to supplying important basic needs to our health system. Regarding preclinical studies, in 2008 the FDA approved the first preclinical test done outside developed countries. The study conducted by Bridge Laboratories was held in China following BPLs. This approval opens an opportunity to start the process of building credibility of institutions located in developing countries. This may include Brazil, considering the progress of its national industry, the number of compounds in the business pipeline, the increased funding in R&D and the degree of diversification of products under development. It is important to emphasize that preclinical testing by GLP is an eliminatory criterion, and this set of rules is fundamental for the quality assessment of the safety and efficacy of new drugs, not only to ANVISA, but to other countries as well, allowing the inclusion of Brazil in the international scene. Finally, it is imperative that INMETRO, ANVISA and RENAMA (National Network of Alternative Methods) go towards harmonization to the international laws. These advances must occur concurrently between pharmaceutical companies, preclinical service providers and regulatory agencies.

